# A review of pears (*Pyrus* spp.), ancient functional food for modern times

**DOI:** 10.1186/s12906-021-03392-1

**Published:** 2021-09-01

**Authors:** Sung-Yong Hong, Ephraim Lansky, Sam-Sog Kang, Mihi Yang

**Affiliations:** 1 College of Pharmacy, Sookmyung Women’s University, Seoul, South Korea; 2 Rimonext Ltd., Haifa, Israel; 3 Pear Research Institute, National Institute of Horticultural and Herbal Science, Rural Development Administration, Naju, South Korea

**Keywords:** Pears, Medicinal function, Detoxification, fiber, Arbutin, Flavonoids

## Abstract

**Background:**

Pears have been world-widely used as a sweet and nutritious food and a folk medicine for more than two millennia.

**Methods:**

We conducted a review from ancient literatures to current reports to extract evidence-based functions of pears.

**Results:**

We found that pears have many active compounds, e.g., flavonoids, triterpenoids, and phenolic acids including arbutin, chlorogenic acid, malaxinic acid, etc. Most of researchers agree that the beneficial compounds are concentrated in the peels. From various in vitro*,* in vivo*,* and human studies, the medicinal functions of pears can be summarized as anti-diabetic,-obese, −hyperlipidemic, −inflammatory, −mutagenic, and -carcinogenic effects, detoxification of xenobiotics, respiratory and cardio-protective effects, and skin whitening effects. Therefore, pears seem to be even effective for prevention from Covid-19 or PM_2.5_ among high susceptible people with multiple underlying diseases.

**Conclusion:**

For the current or post Covid-19 era, pears have potential for functional food or medicine for both of communicable and non-communicable disease.

## Background

Pears (*Pyrus spp*.) have been used as folk medicine and healthy food for more than two millennia [1]. The genus name *Pyrus,* of the family Rosaceae, may be applied for both pears and apples, though *Malus* is the genus name most commonly used for apples, while *Pyrus* (*P.*) is more frequently used for pears. Pears comprise two major types, the ‘European’ or ‘Western’ pears, exemplified by *P. communis*, and the ‘Asian’ pears, typically, *P. pyrifolia* [2], although at least 22 species of pear with over 5000 subspecies or accessions have been recognized [3]. Terms such as ‘Korean pear’, ‘Japanese pear’ and ‘Chinese pear’ are often used interchangeably, but more accurately reflect the geographic milieux from which differing accessions evolved, especially of *P. pyrifolia*, but also from *P. bretschneideri*, *P. sinkiangensis*, and *P. ussuriensis*. Based on evolutionary, morphological and geographical characteristics, pears can be classified as two major accessions, i.e., European and Asian. Asian pears including *P. pyrifolia*, which are round-shaped, possess crisp flesh, high sugar content, especially fructose, low acid content, minimal aroma, and mild flavor, relative to Western or European pears, especially *P. communis*, featuring gourd-shape with soft and smooth flesh, few stone cells, and a stronger aroma and flavor [4]. Among Asian pears, traditional Korean pears, *P. faurie* and *P. serotina,* are smaller than modern cultivars and were developed for local tastes, flavor, and various needs [5–7]. Asian pear cultivars have been reported to contain higher amounts of phenolics, arbutin, and chlorogenic acid than Western pears [8, 9]. In particular, Korean pears hold higher contents of sugar, potassium, and water, relative to Western pears [10]. Scientific names, synonyms, and common names of various pear species are shown in Table 1.

**Table 1:** Summary of scientific names, synonyms, and common names of pear species shown in this review

Scientific name	Synonym	Common name
*Pyrus anatolica*	*Pyrus anatolica* Browicz	Turkey pear
*Pyrus bretschneideri*	**–**	Chinese white pear
*Pyrus calleryana* var. *fauriei*	*Pyrus fauriei* C.K.Schneider	Korean Sun pear
*Pyrus communis*	*Pyrus communis* subsp.	European pear
	*communis*	
*Pyrus malus* var.	*M. domestica* (Suckow)	Ussurian pear, Manchurian
*ussuriensis*	Borkh	pear, Harbin pear
*Pyrus pashia*	*Malus pashia* (Buch.-Ham.	Himalayan pear
	ex D. DON) Wenzig	
*P. pyrifolia*	*Pyrus serotina*	Asian pear
		: Korean pear, Japanese pear
		a part of Chinese pears
*Pyrus sinkiangensis*	**–**	Xinjiang pear

In East Asia including Korea, Japan and China, pears have been used for diverse medicinal applications, e.g., respiratory symptoms relievers, fever managements, inflammation treatment, alcohol hangover, etc. [11, 12]. In particular, pears have been used for digestion of meat, such as a tenderizer in cooking of beef and desserts after consumption of Korean BBQ, Bulgogi. In addition, the traditional function of pears on alcohol hangover was confirmed by recent scientific evidence. That is, in vitro and in vivo studies showed that Korean pears (*P. pyrifolia* cv. Shingo) stimulate main alcohol metabolizing enzymes and eliminates body burden of alcohol and aldehyde [10]. Clinical trials also revealed that the pears alleviate hangover symptoms [13]. Moreover, many researchers have recently found new medicinal functions of pears by diverse studies including chemical analyses, nutritional factors, and in vitro*,* in vivo*,* and human studies [4]. If we further know the traditional functions and local usages of pears, it may help to find out new medicinal functions of pears. Therefore, we conducted a systematic review to provide current information of functional studies of pears, in conjunction with ancient and local applications.

Literature searches were performed from ancient literature to current reports in PubMed at the National Center for Biotechnology Information (NCBI), in Google Scholar, and in Research Information Sharing Service (RISS) at the Korea Education and Research Information Service (KERIS). We extracted evidence-based data of pears for usage, active compounds, and medical function. To obtain reproducibility and avoid publication bias, we collected the information that was confirmed by different researchers as possible as we can.

## Traditional uses of pears

In Korea, pears have been cultivated as a folk medicine and as a sweet fruit since the Samhan period (ca. 300 BCE - 300 CE) [5]. The Taylor–Schechter Genizah collection of a Jewish community mentioned pears in medicinal prescriptions more than one millennium ago [14]. The *Shen Nong Ben Cao Jing* (神農本草經), the first traditional Chinese pharmacopoeia published ca. CE 220, described the uses of pears for relieving fever, quenching thirst, and suppressing cough [1]. The *Ben Cao Gang Mu* (本草綱目) [15], a Chinese pharmaceutical encyclopedia, elaborated on the characteristics of and myriad uses of pears in China, namely that pears are sweet and a little sour, cold, and harmless, however, too much consumption of pears makes people thin and weak and brings out diarrhea. For main treatment, the text goes on: they treat fever, suppress cough, and quench thirst. A slice of a pear is used for relieving pain and preventing decomposition in burn wounds. They are useful for dysarthria caused by irregular fever, stroke, and hypothermia, mitigate fever caused by ague**,** and provide benefits for urination and defecation. They mollify chest tightness, dyspnea, and mental symptoms caused by hyperpyrexia. They further moisturize lungs, cool down the heart, remove sputum, and detoxify abscesses and alcohol poisoning. Pear flowers were said to cleanse dirt on a face, and decoction of pear tree bark provides benefits for seasonal diseases caused by cold weather. The leaves were enlisted for treating scrotal hernia, while pounded leaves extracts were used against mushroom poisoning [15].

**Fig. 1:**
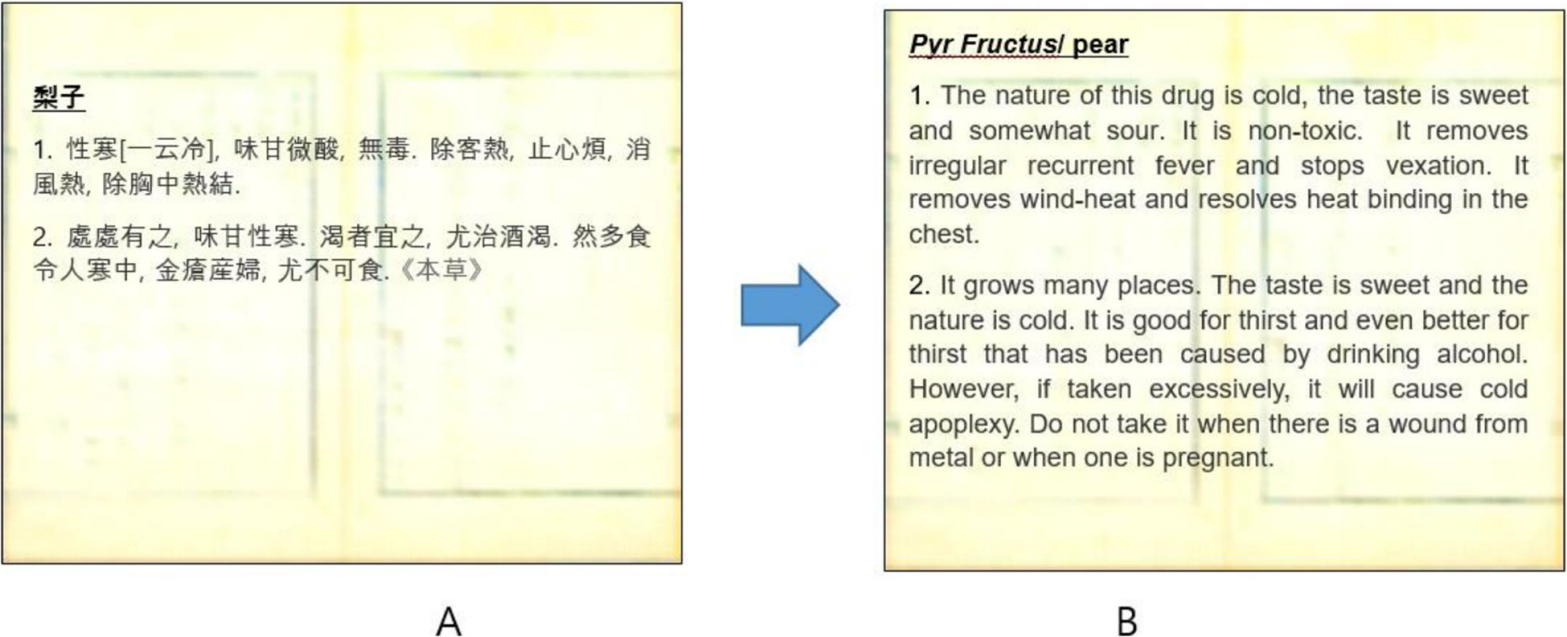
‘DongUiBoGam’ (1613): principles and practice of Eastern medicine, one of memory of the world (UNESCO, 2009), described various usages of Korean pears: **A**, original version; **B**, English translation by Dr. Sang-Woo Ahn of Global DoungUiBoGam Center with minor modification by Mihi Yang

In addition, Jun Heo, a royal physician, described the usage of pears for treating irregular recurrent fever, relieving chest tightness, and quenching thirst, particularly, after drinking alcohol in his Korean traditional medical book, *DongUiBoGam* (東醫寶鑑) [11]. He also depicted the contraindications of pears, e.g., swords cuts or pregnancy, and described that too many pears make the stomach or intestines sick, however, cleaning with water boiled with pear barks provides benefits for scabies and tinea scores (Fig. 1).

## Leading compounds of pears

The major components of pears are water (approx. 80%), sugar and fructose (approx. 15%), and fiber (approx. 2%): Korean pears showed some higher composition of water, sugar, potassium than Western pears (Bartlett: *P. communis*), while Bartlett showed some higher levels of fiber and calcium [10].

**Fig. 2:**
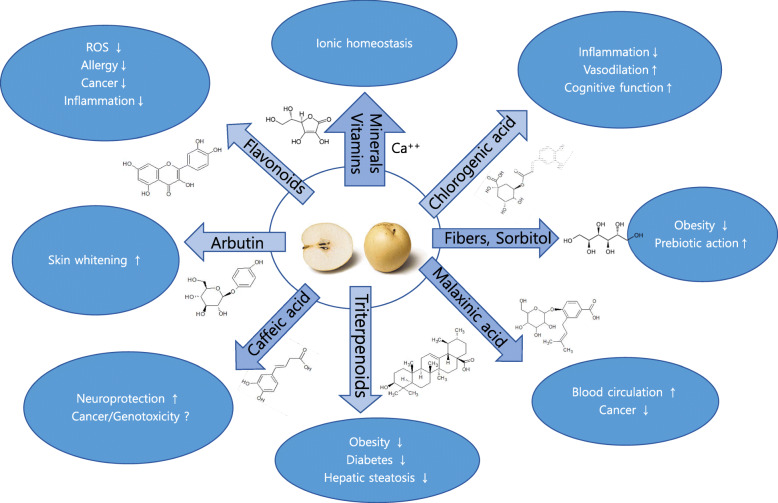
Summary of active compounds and medicinal functions of pears

Various active compounds in pears have been identified, such as polyphenols (phenolic acids, flavonoids), triterpenes, and glucosides [16, 17]. The highest concentration of these phenolic compounds occurs in the leaves, followed by the seeds, peels, and pulps. The phytonutrients in general are richer in peels than the pulp [18, 19]. Pears have thick peels containing pectin, and stone cells with highly thickened, lignified wall of abundant lignin and cellulose [20, 21]. The development of stone cells may be closely related to the synthesis, transfer, and deposition of lignin. As one might expect, the chemical composition in different parts of pears varies. For the monomeric compounds, arbutin, oleanolic acid, ursolic acid, chlorogenic acid, epicatechin, and rutin were reported to be the dominant in various pear cultivars both in peel and in flesh [9]. Figure 2 summerizes the leading functional pear compounds.

### Arbutin

Arbutin, hydroquinone-β-d-glucopyranoside (Fig. 2), is a well-known antibiotic [22], and skin whitening compound [23, 24]. It is degraded into hydroquinone, a skin bleaching agent, and is used in cosmetics as a fragrance, reducing agent, and melanin polymerization inhibitor. Hydroquinone mediates immune function in vitro and in vivo, however, these effects have not yet been established in humans [24, 25]. A Chinese group reported the peel of imported Korean pears (Chinese name, Youran) contained approx. 1.5–20 fold higher amounts of arbutin (6982.0 μg/g dry weight) than other 9 different pear varieties cultivated (323.3–4395.8 μg/g dry weight) in China and South Africa [9]. In Oriental pears, the greatest concentration of arbutin was found in the peel (1.20 mg/g fresh weight), which was 3–5 times greater than that found in the core and 10–45 times greater than the level in the pulp [26, 27]. Therefore, pear skins, especially those of Korean pears, are one of the richest food sources of natural arbutin [28]. As such, arbutin can serve as a potential pear-specific intake biomarker [29].

### Chlorogenic acid

Following arbutin, chlorogenic acid, 5-*O*-caffeoylquinic acid, is the second most abundant phenolic compound (Fig. 2) in pear flesh and peel [30]. In particular, chlorogenic acid reached 106.7–247.5 mg/100 g of fresh weight in immature Korean pears [23]. Chlorogenic acid has been studied and reported to have biological functions such as anti-inflammatory and antioxidant activity [31, 32]. Mechanistic studies on its medicinal function revealed that chlorogenic acid reduces TNF-α, downregulates IL-8 production in Caco-2 cells and RAW264.7 cells, protects neurons, and improves wound healing in vivo*,* suggesting inhibition of inflammation [33, 34]. In addition, chlorogenic acid showed to induce a direct endothelium-dependent vasodilation by increasing NOS, COX, and endothelium-derived hyperpolarizing factor signalling pathways [35]. Recently, a Japanese study showed 6-month intake of chlorogenic acid (330 mg/100 ml of water) significant improved cognitive function from the One Back Test of the Cogstate, the Shifting Attention Test, and Finger Tapping Test as well as in the composite memory, verbal memory, complex attention, cognitive flexibility, executive function, and motor speed domains of the CNS Vital Signs test battery [36]. In addition, chlorogenic acid protected against DNA damage induced by ionizing radiation, suggesting significant radioprotective effects of these compounds [37].

### Caffeic acid

Caffeic acid, one of phenolic acids, is a minor compound in the flesh and peel (56.2 vs. 73.5 mg/kg) of Turkish [22, 24]. Although not structurally related, caffeic acid has been reported to elicit neuroprotective properties with caffeine [38]. Some studies also showed that caffeic acid enhanced collagen production [39, 40]. For colon cancer cells, such as the HCT 15, caffeic acid induced apoptosis, ROS generation and reduction in the mitochondrial membrane potential and showed chemopreventive potential [41].

### Flavonoids

Immature Korean pears contain flavonoids at 182.5–368.9 mg/100 g of fresh weight [23]. B-ring dihydroxylated flavonol derivatives, such as quercetin and isorhamnetin, and monomeric and polymeric flavan 3-ols, such as epicatechin and proanthocyanidins, are dominant among the flavonoids found in ten pears including Radana cultivar [42, 43]. These chemicals have been thought to contribute to color, fruit quality, and plant resistance. In the case of European and Tunisian pear cultivars including the pulp and peel, the predominant flavonoid is (−)-epicatechin (Fig. 2) as terminal and extension units [16].

Following (−)-epicatechin, the anthocyanins, water-soluble pigments composed of an anthocyanidin aglycone, were mainly found in the red skinned pear cultivars [16, 44]. Other flavonoids found in Korean pears (*P. pyrifolia* Chuhwangbae) include quercetin 3-O-glucoside, its aglycone quercetin [18, 45], and dulcisflavan, a catechol [46]. Quercetin 3-O-glucoside is one of the dominant flavonols among leaves and fruits of pears [16].

Flavonoids have been emphasized due to their observed biological effects in vitro, e.g., free-radical scavenging, modulation of enzymatic activity, and inhibition of cellular proliferation, as well as their potential use as antibiotic, anti-allergic, anti-diarrheal, anti-ulcer, and anti-inflammatory, and anticancer agents [47]. However, epidemiologic studies exploring the role of flavonoids in human health have been inconclusive [48].

### Malaxinic acid

Malaxinic acid, 4-(O-β-d-glucopyranosyl)-3-(3′-methyl-2′-butenyl)benzoic *acid,* is a major glucoside in Korean pears [49]. The amounts of malaxinic acid in immature Korean pears reached 0.76—5.86 mg/100 g of fresh weight, although the amounts of malaxinic acid were significantly less, as the pears matured [23]. Several reports have described some medicinal functions of malaxinic acid in Korean pears, e.g., anti-oxidative defense in blood circulation and inhibition of growth of cancer cells, such as BAEC, HT1080, HeLa, and B16/BL6 [23, 50–52]. The isoprenyl side chain in malaxinic acid may contribute to inhibition of a 21–26 kDa protein involved in cancer cell proliferation [53]. Therefore, malaxinic acid can be a candidate for one of major active compounds of pears, however, the evidence should be further collected.

### Triterpenoids

Among triterpenoids, particularly ursolic (Fig. 2), oleanolic, and betulinic acids have been identified in European pear cultivars (*P. communis*), more than 17 fold higher in the peels than flesh (3460.5 ± 1255.9 vs. 201.4 ± 77.1 μg/g of dry weight) [30].

Because of their steroid-like chemical structures, endocrine-related functions can be expected from triterpenoids. For example, ursolic acid showed stimulation of lipolysis in primary-cultured rat adipocytes [54], inhibition of aromatase, which converts androgens into estrogens, and increased energy expenditure, leading to reduced obesity, improved glucose tolerance, and decreased hepatic steatosis [55]. Its isomer, oleanolic acid, has been speculated to have anti-oxidative, anti-tumor, anti-inflammatory, anti-diabetic, and anti-microbial effects [56].

### Other compounds

Plenty of fibers in pears can be prebiotics, which selectively stimulate growth and/or activities of microbial species in the gut microbiota and confer health benefits to the host [57]. For example, pear mixture with carrot, sea buckthorn, plum, or beetroot containing inulin showed growth stimulation in Lactobacillus/Enterococcus [58]. Along with fructose and exceptionally rich fibers, i.e., insoluble cellulose and hemicellulose, and soluble pectin, sorbitol in pears is likely to be responsible for the known laxative properties of pears [1, 30]. Sorbitol, a sugar alcohol, accounts for up to 90% of the total carbohydrate in pears, especially Korean pears. In addition, ascorbic, citric, and malic acids, and minerals, such as magnesium, potassium, calcium, and iron, in pears are likely to support blood pH and ionic homeostasis [16].

## Functional uses of pears

### Anti-diabetic effects

Recent studies have shown that pears possess anti-hyperglycemic effects [59, 60]. Combined apple or other fruits, such as acai, cherry and pear also inhibited diabetic parameters [61, 62]. For example, consumption of apples and pears reduced the risk of Type 2 diabetes mellitus (T2DM) by 18% (95% confidence interval: 0.75–0.88) [62]. In the case of animals, diabetic mice treated with peel extracts of Yaguang pear (*P. ussuriensis* Maxim cv. Yaguang) exhibited significantly lower fasting glucose levels than the diabetic control group, which are possibly related to inhibition of alpha-glucosidase [63]. Similar in vivo results were obtained from the treatment with extracts of immature Asian pears, such as Hosui and Kosui pear cultivars (*P. pyrifolia*) [64]. Furthermore, the diabetic rats, treated with ethyl acetate and ethanol extracts of *P. communis,* exhibited significant reductions in blood glucose compared to diabetic controls and the anti-hyperglycemic effects of pears putatively was due to increased insulin secretion from the pancreatic β-cells [59].

As hyperglycemia is mediated by alpha-amylase and alpha-glucosidase, which promote digestion, absorption, and metabolism of dietary carbohydrates, these enzymes have been pharmacological targets for treating hyperglycemia, because ordinary pharmacological regulators were associated with adverse abdominal effects and poor patient compliance [60, 65]. Pears may serve as a suitable alternative regulating postprandial hyperglycemia by inhibiting alpha-amylase and alpha-glucosidase without any significant side effects. Comparison of 22 different fruit juices in vitro showed that juice extracted from pearple (Chinese white pear, *P. bretchneideri* Rehd.) had the highest inhibition activity against alpha-glucosidase [66]. In vitro examination of extracts of European pear cultivars, Red D’Anjou, Green D’Anjou, Barlett, Bosco, and Comice, also revealed significant inhibition of alpha-amylase and alpha-glucosidase with more potent suppression of alpha-amylase by pulp extracts and more potent inhibition of alpha-glucosidase by peel extracts [65]. Furthermore, an in vitro study with Barlett and Stakrimson pear cultivars showed that the phenolic compounds in Stakimson pear extract might be bioactive and responsible for anti-hyperglycemic property, as it is positively correlated with alpha-glucosidase inhibition [60]. These alpha-amylase and alpha-glucosidase inhibitory activities of pears were observed in many in vitro and in vivo studies and they could provide the foundation for further research on pear extracts aimed to elucidate whether it can play a role in management of T2DM.

The beneficial effects of pear consumption in reducing T2DM risk have been well established in previous observational studies, and the subsequent in vivo and in vitro data have shown a strong positive correlation between pear consumption and improvement in T2DM parameters. Further investigations, particularly studies in humans, might put pears as an ideal phytoceutical option in the management of T2DM patients.

### Anti-obesity activity

Dietary modification and exercise have been recommended for overweight people as beneficial lifestyle interventions. As fruits are low-energy dense and rich in dietary fiber, they can provide stomach satiety with less caloric intake. Specifically, pears have a low energy density of 0.64 kcal/g with plentiful dietary fiber and showed beneficial effects on weight management in a number of different studies [67–70]. For example, the rats on high-fat diets containing pear insoluble dietary fiber (IDF) did not share the same pattern of weight gain as the rats fed diets without pear IDF co-administration and had weights as low as the normal chow-fed group [67]. Therefore, Chang et al. speculated the IDFs extracted from pears exhibited anti-obesity effects, such as acceleration of fat metabolism and reduction of levels of low density lipoprotein –cholestrol (LDL-C) and total cholesterol (TC), by promoting the growth of Bacteriodetes in rats’ gut microbiota. In addition, the pear extract (PE) and *Garcinia cambogia* extract (GE) treated groups showed 4.1 and 14.7% reduction in maturation of pre-adipocytes into adipocytes, respectively, while combined PE and GE synergized to exhibit a 26.9% inhibition, highlighting their potential to prevent weight gain [70].

Some clinical studies have also shown anti-obesity effects of pears. After 12 weeks of daily consumption of fresh pears, green Bartlett or D′ Anjou, leptin concentrations and waist circumference were lower in the pear group than control [71]. Bosc pears decreased the amount of post-exercise exposure to metabolites, leading to improved exercise performance [69]: Pears reduced cortisol levels in the participants by 22% immediately after exercise, promoting faster recovery from strenuous exercises. Many studies support the use of pears for obesity prevention by decreasing caloric intake and promoting exercise.

### Anti-hyperlipidemic effects

As abnormal plasma lipid concentrations are major contributors to CVDs, development of safe anti-hyperlipidemic materials has been desired. Therefore, natural products, such as green tea, onions and garlics, have been studied. Anti-hyperlipidemic effects of pears are particularly observed in hyperglycemic status, since hyperlipidemia is common in diabetic patients. To control lipid levels is even more important in diabetic patients, since they have higher risks of CVDs. Velmurugan and Bhargava found that pear feeding significantly reduced the levels of TC, triglyceride (TG), and LDL-C in hyperglycemic rats, while it increased the level of HDL-C in a dose dependent manner [59]. In addition, the pulp extracts of *P. Communis* L. var. Blanquilla showed the decreased levels of TC by 14.6%, TG by 6.8%, and LDL-C by 17.4% in rats fed cholesterol-containing diets [72]. However, pear peels had more potent lipid-lowering properties than pulp (19.4% for TC, 14.6% for TG, and 33.3% for LDL-C), compared to the control. Therefore, the lipid lowering effects of pears seem to be related to the components such as catechin, which are more condensed in peels than in pulp.

### Anti-mutagenic and -carcinogenic effects

Pears showed some anti-mutagenic and anti-cancer activities by several mechanisms. Firstly, pears can inhibit carcinogenesis of polycyclic aromatic hydrocarbons (PAHs), such as benzo(a)pyrene, which have two main carcinogenic mechanisms, formation of DNA-adducts and production of ROS [73, 74]. We observed that Korean pears reduce benzo(a)pyrene-induced lung cancer in A/J mice, particularly in males [75]. In a biological monitoring study, we also found chemopreventive effects of Korean pears on exposure to PAHs in approx.. 700 Koreans [76]: Urinary concentrations of 1-hydroxypyrene (1-OHP), a major metabolite of PAHs, were analyzed as a biomarker of exposure to PAHs and were significantly decreased in the pear consumers. These results were also confirmed by pharmacokinetic methods in a clinical trial with the subjects who were exposed to PAHs through eating fried chicken with and without pears (*P. pyrifolia* Shingo) [75, 77]: Rapid excretion of urinary 1-OHP was observed in the pear consumers, compared to the non-pear eaters. Therefore, we speculate that Korean pears mediate absorption, distribution, metabolism, and excretion (ADME) of PAHs, particularly excretion of PAHs. The acceleration of PAH excretion may reduce the retention of carcinogens in the pear consumers. In addition, urinary levels of malondialdehyde, a biomarker of lipid peroxidation and oxidative stress, were also decreased in the Korean pear consumers [77, 78]. Considering bioproduction of ROS as another major carcinogenic mechanism of PAHs, we suggest that Korean pears protect PAH-induced oxidative stress. With regard to the botanical characteristics of pear trees, the accumulation of PAHs was also decreased on pear leaves than on other similar-sized plant leaves, such as linden leaves, and the presence of epicuticular waxes on pears may contribute to the low storing of PAHs [76]. Considering PAHs are a main components of PM_2.5_ of air pollution, we suggest pears are effective for prevention from PM_2.5_.

Secondly, pears can be anti-carcinogenic by virtue of their nitrite scavenging activity [79, 80]. As nitrites are widely used during food processing and storage, they may easily react with amines to produce strong carcinogens, such as N-nitrosamines, in the processed food. The nitrite scavenging activity of Asian pears, such as Baekwoon and Niitaka, was 80.7–86.7% [79, 80]. These pears showed comparably high nitrite scavenging activity than other plants, such as onions (50%) or kiwis (75.3–81.8%) [12].

Lastly, there are many functional phytochemicals in pears, such as phenolic compounds including chlorogenic acid and malaxinic acid, which have shown quite diverse anti-carcinogenicity, such as anti-proliferative activities against breast and liver cancer cells [81, 82]. Particularly, Korean pears showed anti-carcinogenic potential due to functions related to mediation of ADME for PAHs, reduction of ROS, nitrite scavenging activity, and antioxidant properties of phenolic compounds.

### Anti-inflammatory effects

Excessive inflammatory responses are a leading cause of non-communicable diseases [83]. However, dietary ingestion of pears, apple, red wine, and strawberries showed inverse associations with inflammation scores (IS) in food-based analyses: Higher dietary anthocyanin and flavonol intakes showed strong association with anti-inflammatory effects in a population of US adults [84]. Anthocyanin intakes reduced IS, such as acute inflammation, cytokines, and oxidative stress, by 73%. Higher intakes of flavan-3-ols, such as catechins, epicatechins, etc., and their polymers were associated with significant reduction in biomarkers of oxidative stress, which included myeloperoxidase, LPL-A2, and isoprostanes, an index of creatinine.

The anti-inflammatory effects of different pear species were compared to those of dexamethasone in carrageenan-induced mice hind paw edema and xylene-induced mice ear edema models [9]. The methanol extracts of pears including *P. ussuriensis* Maxim species (Yaguang) and *P. communis* varieties (Hongpi, Qingpi and Guifei) reduced the mice paw and ear edema to a certain degree and exhibited dose-dependent anti-inflammatory effects. In the case of *P. bretschneideri* Rehd, its ethyl acetate fraction showed the strong inhibition of carrageenan-induced rat paw edema formation and displayed the potent anti-inflammatory activity against xylene-induced ear edema and acetic acid-induced extravasation of Evan’s blue dye at the dose of 200 mg/kg and 400 mg/kg [85]. Triterpenoids and flavonoids, such as 2β,19α-dihydroxy ursolic acid, α-amyrin and quercitrin, were suggested to have these anti-inflammatory effects.

Azuma et al. evaluated the suppressive and anti-inflammatory effects of cellulose nanofibers from Japanese pears (*P. pyrifolia*, Nijuseiki) on inflammatory bowel disease (IBD): The pear nanofiber demonstrated anti-inflammatory effects via suppression of fibroses or by butyrate-mediated inhibition of NF-κB in an IBD murine model [86].

Pear vinegar (PV) also alleviated ulcerative colitis with the disease activity index, which was significantly reduced in rats fed with 9% PV (4.0 ± 0.8), compared to control (7.4 ± 1.4) [87]: Histopathological scoring of severity of tissue damage was also less in the PV treated rats than in controls. PV also suppressed the myeloperoxidase-mediated activation of inflammatory cells and decreased the serum concentration of IL-6. In addition, the leaf extract of *P. ussuriensis* Maxim (Sandolbae) significantly reduced the level of NO in RAW 264.7 cells, as well as IL-6 and IL-1β in TNF-α-induced HaCaT cells [88]. The extract significantly ameliorated the dermatitis severity, scratching tendency, and transepidermal water loss, compared to the negative control among 2,4-dinitrochloro-benzene-treated NC/Nga mice. It normalized skin barriers with decreased production of IgE in mice serum. In addition, some studies showed that arbutin of different pear parts exhibited anti-inflammatory effects on pro-inflammatory cytokines, such as IL-1β, TNF-α, and MCP-1 [9, 89]. These proinflammatory cytokines attribute the cytokine storm of COVID-19. In the absence of an immediate and appropriate therapeutic intervention, COVID-19 patients develop acute respiratory distress syndrome as a result of acute lung damage followed by multi-organ failure and resulting in death [90]. Hence, pears have a strong potential to treat the cytokine storm.

In short, pears showed quite diverse anti-inflammatory effects through suppression of immune responses and this function seems to be related to the combination of diverse chemicals rather than one chemical in pears.

### Respiratory protective effects

A recent systematic review on fruit and vegetable consumption showed that increased intakes of apples and pears was strongly associated with lower incidence of asthma symptoms, less diagnosed asthma, and less bronchial hyper-reactivity, leading these authors to establish an inverse correlation between pear/apple consumption and asthma [91]. Another prospective cohort study concluded that consumption of one or more servings of apples or pears led to significantly lowered risks of chronic obstructive pulmonary disease (COPD) in ex-smokers, with a hazard ratio of 0.70 [92].

In in vitro studies, pears have also shown bronchodilatory effects. Ethanol extracts of ‘*Pyrus pashia* Buch.-Ham. ex D. Don’ exerted a relaxant (0.01–5.0 mg/mL) effect on K^+^ (80 mM) induced contractions in isolated rabbit trachea smooth muscle cells and caused shifting of the Ca^2+^ curves (1.0–3.0 mg/mL) toward right in a manner similar to that of verapamil (3 μM), possibly suggesting presence of Ca^2+^ channel blocking activity [2].

In animal studies, Lee et al. reported that the sensitivity of tracheal smooth muscle of mice to electrical field stimulation and acetylcholine was significantly decreased after treatment with pectins of Asian pear (*P. pyrifolia*): Trachea of mice also showed significantly fewer inflammatory signs, such as thickened bronchial mucosa, loss and/or abnormalities of cilia, lymphocyte proliferation, and sticky mucus plugs along the bronchi. Furthermore, there was 70% reduction in the serum allergen-specific IgE [93]. In addition, another study found that treatment with a combination of extracts from *P. bretschneideri* (pear fruit) and *Fritillaria ussuriensis* (bulb) inhibited tissue edema and reduced vascular permeability, compared to monotherapy with either extract alone among rats [94].

The mechanism by which pears play a beneficial role in the treatment of allergic inflammatory and respiratory diseases such as asthma may be related to their unique combination of polyphenols and flavonoids [2]. Research on flavonoids, including the rutin and quercetin present in pears, indicates some potential activities for the treatment of allergic diseases through the down-regulation of mast cell activation [95].

In addition, Yang et al. (2006) performed a clinical trial with COPD patients using heated Korean pear (*P. pyrifolia* cv. Shingo) juice; however, there was no significant improvement of respiratory health outcomes, including the George’s respiratory questionnaire score or forced expiatory volume for 1 s among COPD patients after 1 month consumption of pear juice [77, 78]. Not heated but unheated pear juice reduced benzo(a)pyrene- induced lung cancer in A/J mice [75]. Therefore, heating for preservation can destroy bioactive compounds such as enzymes in pears.

### Cardio-protective effects

Cardiovascular diseases are the leading global cause of death with 17.9 million mortality events per year [96]. Pear components have shown cardio-protective effects. Concerning active compounds in pear, chlorogenic acid showed to improve ex vivo vessel function and protect endothelial cells against HOCl-induced oxidative damage, via increased production of nitric oxide and induction of Hmox-1 [97].

Among pear species including Red D’Anjou, Green D’Anjou, Bartlett, Bosc and Comice, aqueous pulp extracts of Bartlett showed moderate (18–28%) ACE-I inhibition through in vitro enzyme models, however, no correlation was observed between ACE inhibition and total phenolics or antioxidant capacity [60, 65]. Cardio-protective functions of pears via ACE inhibition were confirmed in vivo systems. However, the mechanisms and active compounds are still obscure.

### Alcohol detoxification and hepato-protection

Pears have been used as a traditional medicine to alleviate hangover symptoms [11]. However, the scientific mechanisms of alcohol detoxification by pears were obscure. Therefore, initial investigations were focused on the effects of pears on ADME of alcohol. The metabolic pathway of alcohol is relatively well-known. Alcohol is mainly metabolized in liver by alcohol dehydrogenase (ADH) into acetaldehyde, a toxic metabolite that increases hepatic lipid peroxidation and oxidative stress [10, 13, 98, 99]. Acetaldehyde is further metabolized by aldehyde dehydrogenase (ALDH) into acetate, which is finally eliminated by the kidney. Several Studies showed that Korean pears (*P. pyrifolia* cv. Shingo) fortified alcohol metabolism by stimulating ADH and ALDH activities in in vitro studies, consequently lowering the levels of blood alcohol or acetaldehyde in in vivo and human studies [10, 13]. In detail, pharmacokinetic analyses showed that Korean pears decreased levels of blood alcohol in *Aldh2* KO mice more significantly than those in normal mice [10], suggesting that the clinical alcohol detoxification effects of Korean pears might be greater in ALDH2 deficient persons than in normal people. Finally, total and average scores of hangover severity were significantly reduced in human subjects who consumed Korean pear juice before alcohol consumption [13].

Korean pears also enhanced the growth of hepatocytes via the increase of ATP synthesis in the cells [100]: Cell proliferation and DNA synthesis in hepatocytes were increased with treatment of [^3^H]-thymidine and pear extracts. Moreover, the pears increased expression of CDK-2 and CDK-4, which are essential for the G1/S transition, but decreased expression of their inhibitors, p21Cip1/CDKNIA and p27Kip1.

For liver, the water extract of pear pomace from *P. pyrifolia* showed suppression of hepatic lipid peroxidation and protection against liver damage in rats fed a high fat/cholesterol diet [101]. In addition, the pear (*P. pyrifolia*) peel extracts significantly prevented the increase of levels of serum alanine aminotransferase and aspartate aminotransferase in acute liver injury among mice [102], mainly showing antioxidant, anti-inflammation, and/or anti-apoptotic properties [103]. When taken together, Korean pears may contribute to prevention of liver from alcohol and non-alcoholic damages.

### Skin whitening effects

Skin whitening is a desirable characteristic in the field of cosmetology, alongside slowing down the aging process and removing wrinkles. As we mentioned above, arbutin was discovered as a skin whitening substance. Pears are a naturally abundant source of arbutin [24, 104]: Skin-lightening action is related to its inhibition of the enzyme tyrosinase, which is critical for generating dark pigments, specifically melanin. Extracts of four cultivars of Korean pears (*P. pyrifolia*)**,** namely Hanareum, Manpungbae, Shingo, and Chuwhangbae, inhibited tyrosinase activity by 50% in melanocytes of mice treated with melanocyte stimulating hormone, α-MSH. It was reported that a high concentration of arbutin was distributed in pear peels [12].

Based on the high contents of arbutin in Korean pears, five unripe Korean pears, i.e., *P. pyrifolia* cultivars, were tested for whitening activities [105]: For whitening activity related to tyrosinase and cellular melanin formation, Manpungbae among the pears showed the strongest tyrosinase inhibition (4.9%) and achieved 74% reduction of the cellular melanin, compared to the non-treated cells. In addition, Yim et al. found that arbutin levels in pear cultivars decreased as the fruit matured. In B16F10 mouse melanoma cells, most of the cultivar extracts inhibited melanin synthesis by about 50% at a 100 μg/mL concentration until 90 days after full bloom [106]. In the case of *P. Anatolica*, which is endemic to Turkey, the leaves and branches showed higher levels of arbutin than fruits (4.74, 4.46, and 0.11%, respectively) [107]. In addition, arbutin-conjugated gold nanoparticles displayed enhanced whitening capabilities, compared to arbutin itself [108].

Protocatechuic acid (PCA) is another phenolic compound with anti-melanogenic and skin-lightening properties in pear peels. PCA significantly suppressed melanogenesis through the inhibition of tyrosinase as well as the co-inhibition of expression of other melanogenesis-related enzymes in mouse melanoma cells treated with Korean pear extracts (*P. pyrifolia* cv. Chuhwangbae) [51].

Given the high amounts of skin whitening agents such as arbutin and PCA, pears, particularly Korean pears, can be a safe and natural source for the therapeutics of hyperpigmentation. In the near future, new pharmaceutical formulation containing pears could be developed.

A summary of medicinal functions of Korean pears and other pears is shown in Table 2.

**Table 2:** Summary of medical functions of Korean pears and other pears

Function	Mechanism^a^	Material	Method	Reference
Antidiabetic	Blood glucose levels ↓	Yaguang (peel extracts); *P. communis*	in vivo	[59, 63]
Anti-obesity	Acceleration of fat metabolism and decreased LDL and TG	Insoluble dietary fiber from pear pomace (China)	in vivo	[67]
	Low calorie intake due to high fiber	*P. communis*	Human	[68]
Alcohol detoxification and hepato-protection	ADH and ALDH ↑	Korean pear (*P. pyrifolia* cv. Shingo)	in vivo	[10]
	Blood alcohol and acetaldehyde ↓	Korean pear (*P. pyrifolia* cv. Shingo)	Human	[13]
	Blockage of lipid accumulation in hepatocytes	Korean pear (*P. pyrifolia* cv. Shingo)	in vitro	[109]
Anti-inflammatory	Fibrosis and inhibition of NFκB ↓	Japanese pear (Nijuseiki)	in vivo	[86]
	MPO-mediated inflammatory cells ↑ serum level of IL-6 ↓	Japanese pear Vinegar	in vivo	[87]
Anti-asthmatic	Suppression of allergic asthma reaction due to reduced serum allergen-specific IgE levels and inflammatory signs	Korean pears (*P. pyrifolia* N. cv. Chuhwangbae)	in vivo	[93]
	Mast cell activation, histamine release ↓	Flavonoids in pears	in vitro	[95]
Bronchodilatory	Ca^2+^ channel blockade in trachea muscle cells	*P. pashia* Buch.-Ham	in vivo	[110]
Anti-hyperlipidemic	Plasma LDL, TG, and Cholesterol ↓; HDL ↑	*P. communis*	in vivo	[59, 72]
Skin Whitening Effects	Formation of cellular melanin ↓	Unripe Korean pears (*P. pyrifolia* cv. Manpungbae)	in vitro	[105]
	Expression of melanogenesis-related enzymes ↓	Korean pear extracts (*P. pyrifolia* cv. Chuhwangbae)	in vitro	[51]
Anti-mutagenic or carcinogenic	Rapid excretion of PAHs	Korean pear (Shingo)	Human in vitro	[75]

^a^*ADH* alcohol dehydrogenase; *ALDH* aldehyde dehydrogenase; *MPO* myeloperoxidase; *LDL* low density lipoprotein; *TG* triglyceride; *HDL* high density lipoprotein; *PAHs* polycyclic aromatic hydrocarbons

## Limitations and methodological suggestions

For comprehensiveness, we have simultaneously tried to review adverse effects of pears. Pears are usually a safely consumed fruit for most people, but there may be some publications biased against pears emphasized as functional foods. With even thorough search, not many reports described pear-related drawbacks. A limited number of reports have addressed carbohydrate malabsorption, such as irritable bowel syndrome and nonspecific diarrhea in infancy and childhood, due to pear or apple juice [111]. Reminding the reader of the contraindication of pears on ‘Ben Cao Gang Mu’ [15] and the high incidence of self-treatment with herbal products among vulnerable populations [112], pears might be avoided for wasting syndrome patients, children, or pregnant women, due to malabsorption. For specific components of pears, the safety of hydroquinone, a metabolite of arbutin, has been concerned after the recognition that benzene caused aplastic anaemia and leukaemia in humans [113]. However, results on hydroquinone from topical, oral, or industrial exposure showed that it is quite safe, compared to the toxicity of benzene. Nevertheless, long term safety was not established, yet.

For other medicinal functions of pears, excretion of urinary stones [114] and wound healing for poor wound healing patients, such as diabetes [115], have been carefully described in some reports as new applications of pears. In addition, a wide plethora of traditional, integrative, complementary and alternative medicines have been touted as the solution for COVID-19, despite the paucity of evidence surrounding the safety and effectiveness of such therapies [116]. Based on the potential of pears as a modulator of pro-inflammatory cytokines via various anti-oxidative flavonoids [117], evidence-based studies of pears are needed for a new application, e.g., prevention and treatment of new communicable diseases.

## Conclusions

**Fig. 3:**
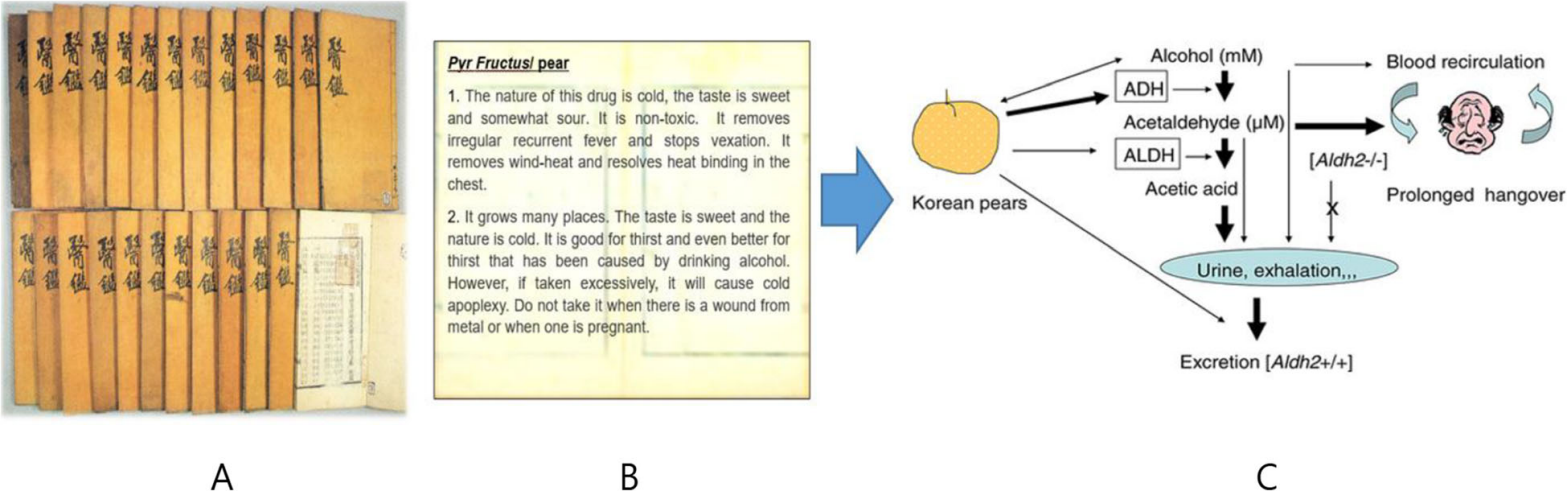
New medicinal function of pears from the old: **A**, DongUiBoGam; **B**, medicinal uses and toxicity warning of pears in DongUiBoGam; **C**, potential mechanisms of Korean pears on alcohol detoxification [10]

For medicinal functions of pears, we learn from the old and make it a new (Fig. 3). Traditional functions of pears have been gradually confirmed with scientific evidence. Pears, an old and new fruit, show beneficial effects on various degenerative diseases and have a strong potential as functional food or medicine for high susceptible people with underlying diseases to prevent from new communicable diseases in the current or post COVID-19 era.

## Data Availability

The datasets supporting the conclusions of this article are included within the manuscript.

## Abbreviations

ADH: Alcohol dehydrogenase
ADME: Absorption, distribution, metabolism, and excretion
ALDH: Aldehyde dehydrogenase
COPD: Chronic obstructive pulmonary disease
GE: *Garcinia cambogia* extract
HDL-C: High density lipoprotein- cholesterol
IBD: Inflammatory bowel disease
IDF: Insoluble dietary fiber
IS: Inflammation scores
LDL-C: Low density lipoprotein- cholesterol
1-OHP: 1-hydroxypyrene
*P.*: *Pyrus*
PAHs: Polycyclic aromatic hydrocarbons
PCA: Protocatechuic acid
PE: Pear extract
PV: Pear vinegar
T2DM: Type 2 diabetes mellitus
TC: Total cholesterol
TG: Triglyceride
